# Skeletal Muscle-Derived Human Mesenchymal Stem Cells: Influence of Different Culture Conditions on Proliferative and Myogenic Capabilities

**DOI:** 10.3389/fphys.2020.553198

**Published:** 2020-09-16

**Authors:** Stefano Testa, Carles Sánchez Riera, Ersilia Fornetti, Federica Riccio, Claudia Fuoco, Sergio Bernardini, Jacopo Baldi, Marco Costantini, Maria Laura Foddai, Stefano Cannata, Cesare Gargioli

**Affiliations:** ^1^Department of Biology, University of Rome Tor Vergata, Rome, Italy; ^2^IRCCS Regina Elena National Cancer Institute, Rome, Italy; ^3^Institute of Physical Chemistry, Polish Academy of Sciences, Warsaw, Poland

**Keywords:** skeletal muscle, mesenchymal stem cells, myogenic differentiation, human serum, platelet-rich plasma

## Abstract

Skeletal muscle tissue is characterized by restrained self-regenerative capabilities, being ineffective in relation to trauma extension both in time span (e.g., chronic diseases) and in size (e.g., large trauma). For these reasons, tissue engineering and/or cellular therapies represent a valuable solution in the cases where the physiological healing process failed. Satellite cells, the putative skeletal muscle stem cells, have been the first solution explored to remedy the insufficient self-regeneration capacity. Nevertheless, some limitation related to donor age, muscle condition, expansion hitch, and myogenic potentiality maintenance have limited their use as therapeutic tool. To overcome this hindrance, different stem cells population with myogenic capabilities have been investigated to evaluate their real potentiality for therapeutic approaches, but, as of today, the perfect cell candidate has not been identified yet. In this work, we analyze the characteristics of skeletal muscle-derived human Mesenchymal Stem Cells (hMSCs), showing the maintenance/increment of myogenic activity upon differential culture conditions. In particular, we investigate the influence of a commercial enriched growth medium (Cyto-Grow), and of a medium enriched with either human-derived serum (H.S.) or human Platelet-rich Plasma (PrP), in order to set up a culture protocol useful for employing this cell population in clinical therapeutic strategies. The presented results reveal that both the enriched medium (Cyto-Grow) and the human-derived supplements (H.S. and PrP) have remarkable effects on hMSCs proliferation and myogenic differentiation compared to standard condition, uncovering the real possibility to exploit these human derivatives to ameliorate stem cells yield and efficacy.

## Introduction

The self-regenerative process of skeletal muscle tissue is a complex phenomenon that engages several types of resident and circulating stem cells with different potentialities (Yin et al., 2013; Čamernik et al., 2018). Among all of these cell types, the most important involved in the repairing process are satellite (Mauro, 1961) and non-satellite cell populations (Crisan et al., 2008; Sacchetti et al., 2016). While the former has been well characterized in the last decades and their activation mechanism unraveled relying on satellite cell niche regulation by the complex interaction among Notch, Collagen V, and Calcitonin receptor (Baghdadi et al., 2018), the latter are gaining increasing interest in the research community thanks to readiness of isolation and expansion, and last but not least the better migratory capacity (Rando et al., 1995; Ferrari et al., 1998; Sacco et al., 2008).

Notably, mesenchymal stem cells (MSCs) are a heterogeneous population composed of satellite and non-satellite cells, the latter including interstitial cells called PW1^+^/Pax7^−^ interstitial cells (PICs; Mitchell et al., 2010), fibro-adipogenic progenitors (FAPs; Uezumi et al., 2010), muscle side population cells (SP), and muscle resident pericytes (Gussoni et al., 1999; Kumar et al., 2017).

MSCs were initially described in the bone marrow as bone marrow-derived mesenchymal stromal/stem cells (BM-MSCs) for their unique combination of features, which include fibroblast-like morphology, clonogenicity, multipotency, and *in vitro* adherence on plastic surface unlike the hematopoietic counterpart (Friedenstein et al., 1970; Pittenger et al., 1999). Cells with similar *in vitro* abilities have been isolated from numerous adult tissues and organs, including skeletal muscle, both from small mammals and human biopsies (Hass et al., 2011; Čamernik et al., 2018). So far, the most popular and studied tissue source employed for MSC isolation has been the bone marrow thanks to its availability and accessibility in the human body. Nevertheless, over the last decade other tissue sources have been explored such as fat tissue, umbilical cord, dental pulp, skin, placenta, and even brain (Yamada et al., 2010; Appaix, 2014; Orciani et al., 2014; Li et al., 2015; Bieback and Netsch, 2016; Pelekanos et al., 2016; Amati et al., 2017). Despite heterogeneity primarily due to different isolation tissue sources, MSCs maintain characteristic expression markers such as CD90, CD44, CD73, CD29, and CD105 while missing the hematopoietic ones such as CD34, CD45, and CD11 (Haynesworth et al., 1992; Lodie et al., 2002; Suva et al., 2004). Besides, they retain and share similar differentiation potential in mesoderm-lineage tissues including bone, fat, cartilage, and skeletal muscle (Okamoto et al., 2002; Sottile et al., 2002; Zhang et al., 2009; Almalki and Agrawal, 2016; Kozlowska et al., 2019).

As regards skeletal muscle tissue, BM-MSCs differentiate into myogenic lineage exclusively upon exposure to demethylating agent 5-azacytidine (Wakitani et al., 1995; Jackson et al., 2007) or in co-culture with myocytes (Lee et al., 2005). More recently, Sacchetti and collaborators have demonstrated that skeletal muscle-derived MSCs have, beyond an intrinsic heterogeneity, spontaneous and important myogenic capabilities *in vitro* and *in vivo*. Moreover, the authors have shown that the differentiation potentiality may vary radically according with the tissue origin and that MSCs immune-profile does not reflect identical cells and function (Sacchetti et al., 2016). Human MSCs (hMSCs), due to their multiple potentialities, could still represent a good candidate for cell therapy (De Bari et al., 2003; Oppermann et al., 2014; Klimczak et al., 2018; Pittenger et al., 2019). Hence, in order to translate MSCs into the actual clinical scenario, researchers still need to address a long-standing challenge: to produce *in vitro* a clinically relevant number of cells without affecting their differentiation capacity. Moreover, culture conditions should be standardized to fulfill good manufacturing practice (GMP) protocols and to avoid possible contamination or immunological reactions due to xenogeneic medium supplement, e.g., animal derived sera (Tonti and Mannello, 2008). In fact, as demonstrated in numerous studies, the expansion of hMSCs strongly depends on the culture conditions, being anchorage-dependent and requiring medium supplemented with 10–20% serum (De Bari et al., 2003; Meuleman et al., 2006; Čamernik et al., 2018; Musiał-Wysocka et al., 2019). Additionally, interactions among cells, growth surface, and surrounding medium influence many aspects of cell behavior, such as efficiency of isolation, proliferation rate, maintenance in culture, stemness, and differentiation potentiality (Kozlowska et al., 2019; Musiał-Wysocka et al., 2019).

Given all these key aspects, over the past years a growing interest has been focused on biologic agents such as platelet-rich plasma (PrP) to complement the cell culture medium and/or significantly ameliorate musculoskeletal tissue healing (Hamid et al., 2014; Kunze et al., 2019). However, their real beneficial effect is still questioned (Grassi et al., 2018).

In order to shed some light on the potentialities of skeletal muscle-derived hMSCs for skeletal muscle regeneration, here we compare different culture conditions evaluating immunophenotypical aspect, cell growth, and myogenic differentiation capability. The obtained results show the possibility to ameliorate hMSCs proliferation and differentiation capabilities adding human-derived supplements (H.S. and PrP) to basic medium or employing enriched growth medium (Cyto-Grow) for human stem cells culturing.

## Materials and Methods

### Isolation of Mesenchymal Stem Cells From Muscle Biopsies and Cell Culture and Differentiation

hMSCs were isolated from skeletal muscle tissue using a protocol that includes mechanical mincing, enzymatic digestion with type II collagenase, filtration, and selection of the colonies on plastic surface at low confluence (Sacchetti et al., 2016; Vono et al., 2016). Briefly, human skeletal muscle biopsies were finely minced with a surgical knife and collected in a solution of collagenase type II (100 U/ml in PBS Ca^2+^/Mg^2+^), subsequently left to incubate in a thermal shaker for 45 min at 37°C. After digestion, the solution was centrifuged at 300 *g* for 10 min at room temperature (RT) and then aspirated without disturbing the pellet. The pellet was then resuspended in 15 ml of PBS and filtered through progressively finer cell strainers: 100, 70, and 40 μm. Cells were counted with a Burker counting chamber and plated on conventional Petri dishes (BD Falcon) at low confluence (10^3^ cells/cm^2^) to promote the growth of cells that had clonogenicity and therefore stem potentiality. The freshly isolated MSCs were divided into two experimental groups respectively cultured in either α-MEM (Gibco) supplemented with 20% heat-inactivated fetal bovine serum (FBS, EuroClone), penicillin (100 IU/ml, Gibco) and streptomycin (100 mg/ml, Gibco), or Cyto-Grow medium (Resnova) supplemented with penicillin (100 IU/ml, Gibco) and streptomycin (100 mg/ml, Gibco). In both conditions, cells were cultured at 37°C and 5% CO_2_ and left to incubate for 15 days, the time required for colonies formation. After colonies formation, cells were expanded for both flow cytometric and differentiation analysis. In particular, differentiation was achieved spontaneously once the culture reached a cell confluence of 80%, without needing differentiation medium or specific factors. Alternatively, freshly isolated cells and colonies were harvested and used for flow cytometry analysis.

### Flow Cytometry Analysis

The 1 × 10^6^ cells for each experimental condition were harvested with Lonza™ Trypsin-Versene™-Trypsin-EDTA (Fisher Scientific, #BE17-161E), resuspended and centrifuged at 300 *g* for 10 min at RT. Cells were washed twice with PBS supplemented with BSA 0.5% (Bovine Serum Albumin, AppliChem, #A1391) and EDTA 2 mM at 300 g for 10 min at 4°C. Samples were then resuspended in PBS and incubated with APC-A700 anti-human CD56 (N901, #B92446 Beckman Coulter), APC-A750 anti-human CD90 (Thy-1/310, #B36121 Beckman Coulter), and Vioblue anti-human CD45 antibodies (REA747, #5180719178 Miltenyi Biotec) for 30 min at 4°C. After incubation, cells were washed in PBS, centrifuged at 300 *g* for 10 min at 4°C, and resuspended in PBS. Samples were visualized on Cytoflex S, 3 lasers (488, 405, and 638 nm), and 13 detectors (Beckman Coulter). Live cells were gated based on side scatter and forward scatter. Data were analyzed by CytExpert software (Beckman Coulter).

### Growth Curves

Growth curves were obtained using 48-well plates containing 2 × 10^4^ cells/well. Three wells of the 48-well plates were prepared for every experimental point (2, 5, 7, and 9 days) and every experimental condition: (i) α-MEM supplemented with 20% FBS (control), (ii) 5% human serum (low-H.S.), (iii) 10% human serum (medium-H.S.), (iv) 20% human serum (high-H.S.), (v) 5 × 10^5^ platelets/ml (low-PrP), (vi) 1 × 10^6^ platelets/ml (medium-PrP) and 1.5 × 10^6^ platelets/ml (high-PrP). Human serum (H.S.) and PrP were provided by Dr. Foddai. Serum was obtained from a sample of whole blood donor (ranging from 30 to 60 years old). Ten milliliter of venous blood is yielded without addition of anticoagulant, and each blood sample is prepared by centrifugation (5’ at 3000 RPM and 15°C) to separate red blood cells from serum. The obtained serum was transferred to an empty sterile tube and criopreserved at −80°C.

The PrP was a cell concentrate of leucodepleted platelets suspended in plasma, obtained from single donor of blood subjected to a platelet apheresis procedure using a Fresenius Kabi Amicus cell separator. An aliquot of cell concentrate is transferred into a sterile falcon and brought to different concentration: 0.5 × 10^5^/ml, 1.0 × 10^5^/ml, and 1.5 × 10^5^/ml. Cellular proliferation was evaluated by harvesting cells at each time point and scoring the media in a Burker counting chamber.

### Immunofluorescence Analysis

Cells were fixed with 4% PFA in PBS for 10 min at 4°C and processed for immunofluorescence analysis as previously described (Testa et al., 2017). Briefly, cells were washed with PBS and blocked with 10% goat serum in PBS for 1 h at RT. Subsequently, cells were incubated with the primary antibody anti-myosin heavy chain (MF20, mouse monoclonal, DHSB, diluted 1:2) or anti-ki67 (rabbit polyclonal, Novus Biologicals #NB110-89717, diluted 1:200) for 1 h, followed by incubation with Alexa Fluor 555-conjugated goat anti-mouse IgG (H + L; Thermo Fisher Scientific #A21422, diluted 1:400) and 488-conjugated goat anti-rabbit IgG (H + L; Thermo Fisher Scientific #A11008, diluted 1:400) for 1 h. Finally, nuclei were stained with 300 nM DAPI (Thermo Fisher Scientific) for 10 min. Specimens were viewed using a Nikon TE 2000 epifluorescence microscope equipped with a Photometrics Cool SNAP MYO CCD camera.

### Statistical Analysis

All experiments were performed in biological and technical triplicate (*n* = 9). Data were analyzed using GraphPad Prism 7, and values were expressed as means ± standard error (SEM). Statistical significance was tested using either ONE WAY ANOVA and Tukey’s *post hoc* test or *t*-test when only two parameters were compared. A probability of less than 5% (*p* < 0.05) was considered to be statistically significant.

## Results

### Influence of Different Culture Media on CD90 Expression, Proliferation, and Myogenic Capabilities of Skeletal Muscle-Derived hMSCs

**Figure 1:**
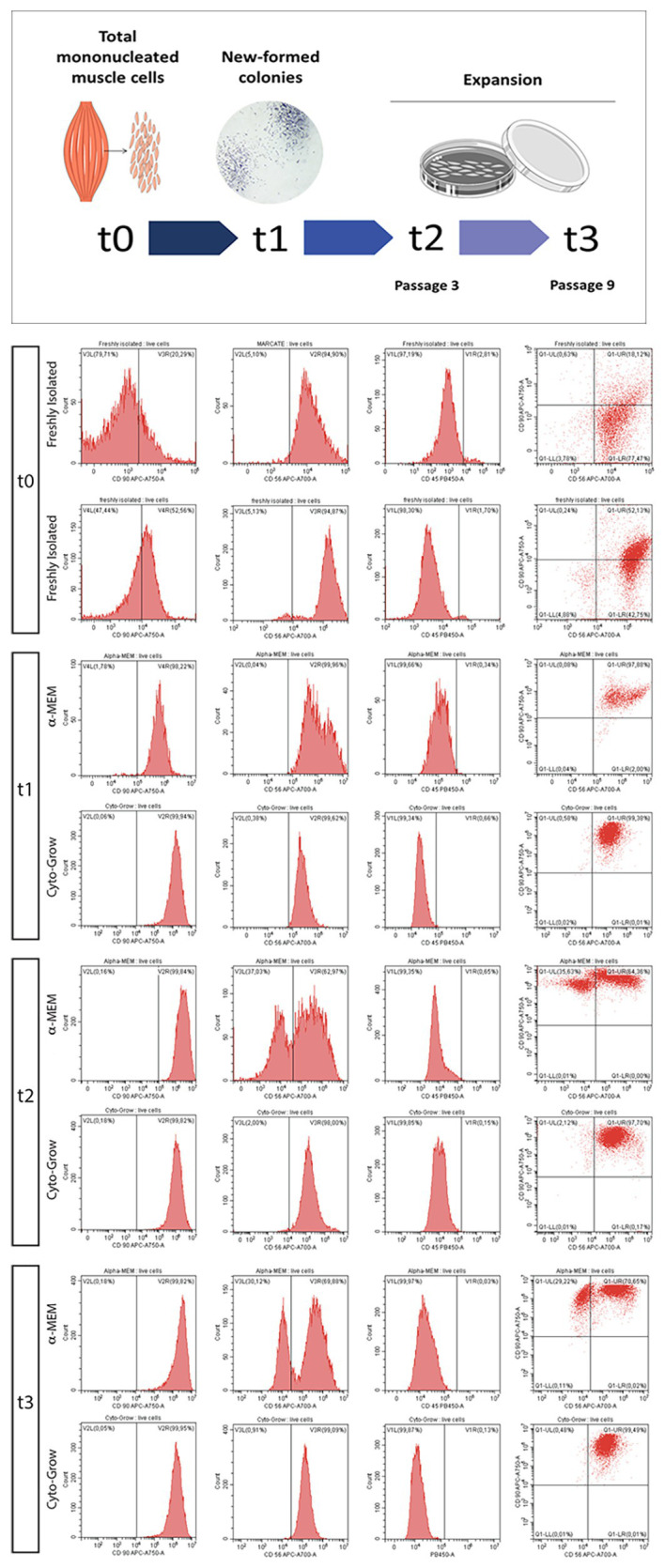
Human MSC (hMSC) characterization by flow cytometric analysis. Scheme representing the time points analyzed in the flow cytometric analysis, from skeletal muscle-derived hMSCs isolation up to late doubling time: total mononucleated cells freshly isolated from the tissue (t0), new formed colonies after 15 days (t1), expansion passage 3 (t2), and expansion passage 9 (t3). Flow cytometry analysis for CD56, CD90, and CD45 in relation to different culture media: α-MEM and Cyto-Grow, showing the histograms of the signal for each antibody used and the dot plot displaying the double positivity for CD90 and CD56.

Mononucleated cells from human derived skeletal muscle biopsies were examined at different time points by flow cytometry analysis in order to test whether different culture media would affect cell populations heterogeneity and behavior. In this regard, hMSCs were divided in two experimental groups according to the differential culture conditions: (i) α-MEM supplemented with 20% of FBS (standard medium) or (ii) Cyto-Grow (rich medium). The two experimental groups were analyzed at different time points (t0–t3) starting from isolation up to late doubling time, namely: total mononucleated cells soon after isolation from fresh muscle biopsy (t0), colonies formation stage according to MSCs conduct (after 15 days on standard plastic culture; Sacchetti et al., 2007, 2016; t1), expansion passage 3 (t2), and expansion passage 9 (t3; Figure 1). Cells were, accordingly, analyzed by flow cytometry analysis to evaluate the expression of hMSCs stemness marker CD90 as indicated by Kisselbach and colleagues (Kisselbach et al., 2009) and the human muscle marker CD56 (labeling human myogenic cells), further using CD45 as a negative marker (hematopoietic compartment). Analysis at t0 revealed a heterogeneous population of mononucleated cells, presenting several stem cells CD90^+^ (15.75%) together with abundant myogenic cells CD56^+^ (76.65%) displayed in the dot-plot, besides hematopoietic stem cells positive for CD45 (11.45%; Figure 1, t0). Hence, t0 population has been split and cultured in two different media (α-MEM or Cyto-Grow, used in the following stages) revealing differences in terms of marker expression already at t1. Here, the colonies formed in standard medium presented the tendency to segregate in two subpopulations: one exclusively CD90^+^ and the other one double positive CD56^+^/CD90^+^. Differently, cells cultured in rich medium formed a more homogeneous double positive population CD56^+^/CD90^+^ (Figure 1, t1). At t2, in standard medium, two well distinct cell subpopulations became more evident reaching at t3 29.22% of CD90^+^ cells and 70.65% of double positive CD56^+^/CD90^+^ cells, while expansion in rich medium selected myogenic CD56^+^ cell population at the expenses of CD90^+^/CD56^+^ double positive one (Figure 1, t2 and t3). The CD45^+^ hematopoietic stem cells are lost on both groups already at t1 (Figure 1).

**Figure 2:**
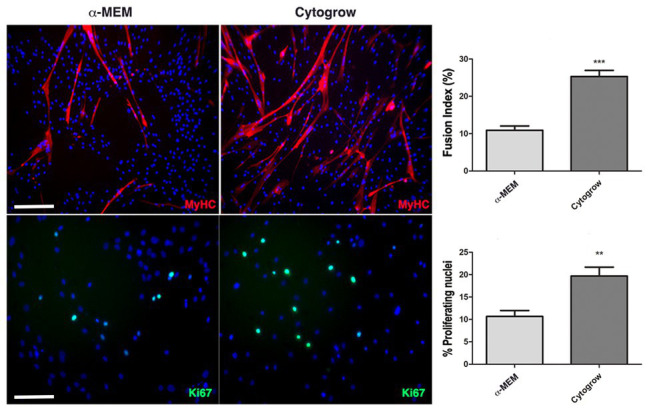
Culture conditions influence cell behavior. Immunofluorescences against MyHC (red) and Ki67 (green) reveal the remarkable differences in terms of differentiation and proliferation employing Cyto-Grow commercial medium compared with α-MEM (nuclei labeled in blue by DAPI). The respective quantifications are reported as fusion index and rate of proliferating nuclei (Ki67 positive). Statistical analysis: one-way ANOVA and Tukey’s test. ^**^p < 0.01, ^***^p < 0.001 (*n* = 3). Scale bars: MyHC = 200 μm; Ki67 = 100 μm.

To evaluate the influence of the two different media on proliferation rate and myogenic differentiation, immunofluorescence analysis against the proliferation marker Ki67 and the muscle terminal differentiation marker myosin heavy chain (MyHC) has been performed on both experimental groups. Results show a significative increase of Ki67^+^ nuclei in rich medium grown cells (Figure 2) implying a higher proliferation rate. Moreover, in both media a spontaneous myogenic capability was observed in skeletal muscle-derived hMSC culture with a higher efficiency in rich medium (Figure 2). This evidence was confirmed by fusion index analysis, a quantitative indicator of the cell differentiation level that is calculated by dividing the number of nuclei present in the myotubes (threshold: 3 nuclei per myotube) by the number of total nuclei (Figure 2).

### Employment of Different Human Serum Concentration as Medium Supplement for hMSCs Culturing

**Figure 3:**
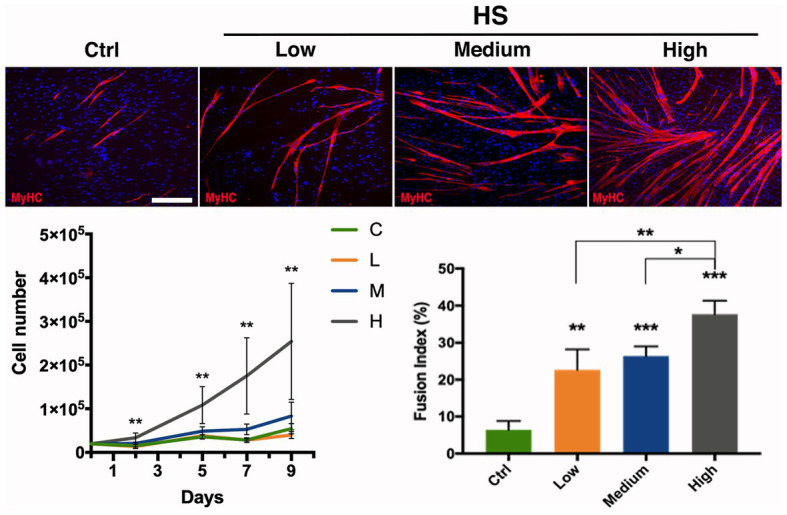
Effect of human serum on hMSC proliferation and differentiation. hMSC proliferation and differentiation analyzed upon different medium supplementations exposure. α-MEM medium added with: Ctrl (20% FBS), Low (5% Human Serum), Medium (10% Human Serum), and High (20% Human Serum). Anti MyHC (red) immunolabeling on hMSCs cultured in different conditions as indicated; nuclei were marked with DAPI (blue). Cell growth is indicated as proliferation curve evaluated up to 9 days. Myogenic evaluation relative to MyHC signal is represented as fusion index at day 9 of culturing in the investigated conditions. Statistical analysis: one-way ANOVA and Tukey’s test. ^*^p < 0.05, ^**^p < 0.01, ^***^p < 0.001 (*n* = 3). C–F: scale bar 250 μm.

In order to test whether human serum could be employed as medium supplement for hMSCs growth and thus satisfy the need of MSC culture standardization avoiding animal derivatives, different concentration of this compound has been used to investigate its efficacy. Therefore, hMSCs have been cultured in α-MEM supplemented with 20% FBS as standard (control) medium, or at increasing concentration of human serum: low (5%), medium (10%), and high (20%). At different time points (2, 5, 7, and 9 days of culture) the cells were harvested to score proliferation rate. hMSCs growth revealed a significant dependence on human serum concentration: in particular, low concentration (5%) of human serum was comparable to the control medium (20% FBS) growth rate, while already at medium concentration (10%) it was possible to observe a remarkable enhancement in the hMSCs proliferation, reaching a statistically significant increment at high concentration (20%) in every analyzed time point up to a more than 5-fold increase at day 9 (Figure 3).

Myogenic differentiation has been assessed by MyHC expression by means of immunofluorescence analysis at culturing day 9, a suitable time allowing cells to fuse and differentiate into myotubes. A clear trend was also observed for the number of formed myotubes, with the highest number of myotubes obtained with the highest human serum concentration (Figure 3). These observations have been further confirmed by the fusion index analysis showing the effect of human serum enhancing significatively myogenic differentiation, revealing the higher concentration being more efficient to the control (4-fold increase) and the other two concentrations (Figure 3).

The effect of human serum supplementation on hMSCs heterogeneity has also been evaluated. Flow cytometry assay was performed employing the higher H.S. concentration tested in the previous experiments, indicating the formation of a homogeneous myogenic population CD90^+^/CD56^+^ double positive (Supplementary Figure 1).

### Influence of Different Platelet-Rich Plasma Concentrations on hMSCs Proliferation and Differentiation

**Figure 4:**
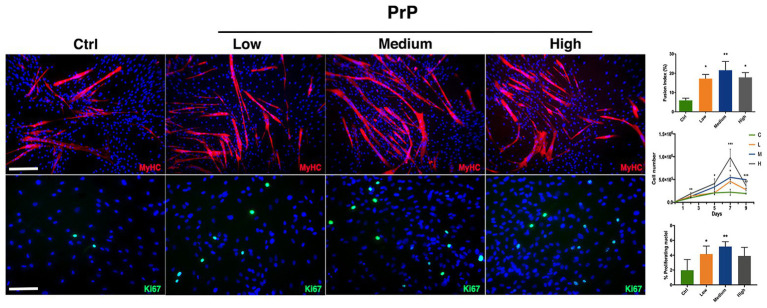
platelet-rich plasma (PrP) affects hMSC proliferation and myogenesis. Immunofluorescences against MyHC (red) and Ki67 (green) revealing differences in proliferation and differentiation in relation to different PrP concentration. α-MEM supplemented with: Ctrl (20% FBS), Low (5 × 10^5^ platelets/ml), Medium (1 × 10^6^ platelets/ml), and High (1.5 × 10^6^ platelets/ml); nuclei were counterstained in blue by DAPI. The relative quantification is reported as fusion index for muscle differentiation, proliferating curve, and rate of Ki67 positive nuclei for cell doubling. Statistical analysis: one-way ANOVA and Tukey’s test. ^*^p < 0.05, ^**^p < 0.01, ^***^p < 0.001 (*n* = 3). Scale bars: MyHC = 200 μm; Ki67 = 100 μm.

In order to test PrP effect on cell division rate and myogenic differentiation, hMSCs have been cultured with low (5 × 10^5^ platelets/ml), medium (1 × 10^6^ platelets/ml), and high (1.5 × 10^6^ platelets/ml) concentrations of PrP as medium supplement. To evaluate the proliferation under PrP influence, cells were harvested and counted at 2, 5, 7, and 9 days upon PrP exposure. The resulting growth curves revealed that medium supplementation with high concentration of PrP significantly increased cell division for 7 days, while a decrease was observed at day 9 (Figure 4). The other concentrations of PrP demonstrated a relative moderate and progressive increase up to day 7, reaching the plateau at day 9. The PrP effect on hMSCs proliferation has been further confirmed by immunofluorescence analysis for Ki67 at day 9 (Figure 4). The obtained results, plotted scoring Ki67^+^ nuclei, were consistent with the cell growth curves showing a raise of proliferating nuclei at every PrP concentration compared to the control, being significant at low and medium concentrations (Figure 4). As regards the PrP effect on myogenic differentiation, fusion index assay was performed revealing a significative increment of myotube formation as a consequence of PrP addition, despite the fact that the effect is not concentration-dependent in a significative way (Figure 4). Moreover, the cell population heterogeneity exposed to the higher dose of PrP tested was assessed by flow cytometry assay, yielding a homogeneous and myogenic population CD90^+^/CD56^+^ double positive (Supplementary Figure 1).

## Discussion

Today, one of the most challenging tasks in skeletal muscle regenerative medicine is to find a suitable stem cells source for a large and easy cellular expansion, avoiding losing the myogenic potential (Errico et al., 2018). In fact, satellite cells, despite being the effective muscle stem cells, present a low isolation rate and can be kept in culture only for a few passages without losing myogenic potentiality. This has prompted researchers to investigate other stem cell populations (Fuoco et al., 2016a,b). However, despite the efforts of the last decades leading to the characterization of several stem cell populations with mesenchymal origin and promising myogenic potentiality, obtaining a large amount of human myogenic primary cells for tissue engineering or cell therapy approaches is still an unmet need (Torrente et al., 2007; Skuk et al., 2014; Sacchetti et al., 2016). For this reason, in this work we evaluated how culture conditions can modulate different basic features of skeletal muscle-derived hMSCs, such as proliferation rate, myogenic potential efficacy, and maintenance.

Here, we tested whether different growth media can directly influence hMSCs – freshly isolated from human skeletal muscle biopsies – heterogeneity, expansion potentiality, and differentiation capabilities. In particular, we employed different culture conditions comprising different media (α-MEM and Cyto-Grow) and different supplements (Human Serum and PrP). To monitor the effect of the two different media on hMSCs heterogeneity, we performed a time course analysis by cytofluorimetric assay, investigating CD90 positivity, a MSCs marker, and myogenic potential (CD56 positivity). The obtained results revealed that both media were able to maintain the positivity to the stem cell marker CD90 for many doubling times (up to passage 9). Interestingly, while in standard medium (α-MEM + 20% FBS) we observed the formation of two distinct subpopulations (single positive CD90^+^ and double positive CD90^+^/CD56^+^), in the rich medium a more homogeneous population double positive was formed and maintained, showing to directly promote the maintenance of the myogenic properties right from the early stages. Thus, in order to obtain a large number of myogenic stem/progenitor cells, the use of a rich medium – such as Cyto-Grow – would ameliorate remarkably the isolation and expansion procedures. Moreover, Cyto-grow proved to be a better medium choice for promoting proliferation and a robust myotubes formation compared to standard medium, as confirmed by Ki67^+^ nuclei scoring and fusion index assay.

In parallel, we investigated the possibility to employ human-derived medium supplements in order to avoid animal derivatives and then guaranteeing completely species-specific cell condition, the “humanizing supplement” (Roberts et al., 2012; Shanbhag et al., 2017). These supplements were employed to replace animal derived serum to the standard medium (α-MEM + 20% FBS) and then to identify the best in terms of CD90 expression maintenance *in vitro*, proliferation, and differentiation ability in relation to what is considered the standard condition in literature (Shahdadfar et al., 2005). Hence, we tested different human serum concentration (ranging from 5 to 20%), observing that at higher concentration (20%) the hMSCs proliferation rate was about 5-fold greater than control (20% FBS). The medium dose (10%) had just a slight effect, while the lower concentration (5%) was comparable with the control in terms of proliferation rate. These results demonstrated the possibility to exploit human serum to ameliorate human cell proliferation rate and then to reduce the cellular expansion time. This strategy has a great clinical potential as it avoids the use of animal-derived supplements and, on the other hand, lays the basis for the development of a culturing protocol that fulfill GMP regulations essential for human cell clinical application. Additionally, we have also investigated the impact of the human serum on hMSCs myogenic capabilities, showing – upon 9 days of culture – a noteworthy enhancement of the differentiated myotubes in relation to the serum concentration, with a significant increment (up to 4-fold increase) of the fusion index at high concentration (20%).

Taken together these results suggest a strong direct correlation between human cell physiological processes, such as proliferation and differentiation, and medium supplementation with increasing doses of a pool of human sera. Interestingly, unlike what was reported by Shahdadfar et al. (2005), we observed that the beneficial effect of the human serum is independent from the autologous donor derivation. This divergence could rely on the different MSC isolation origin skeletal muscle versus bone marrow (Shahdadfar et al., 2005).

Furthermore, we tested PrP action on hMSCs to exploit another humanizing medium supplement largely used in clinical therapeutic approach for pathologies affecting the musculoskeletal system (Roberts et al., 2012; Shanbhag et al., 2017). In this case, we observed that cell proliferation rate increases with the increase of PrP concentration, reaching the maximum at day 7 for the highest PrP concentration (approx. 4-fold increase compared to the control), further confirmed by Ki67 expression quantification (Figure 4). Nevertheless, at day 9 we noticed a dramatic decrease in the proliferation rate, with a more pronounced effect for the highest dose of PrP, probably due to the excessive over-confluence compromising cell health. PrP addition also promoted increased myotubes formation, although dose-independent.

Finally, we have evaluated the effect of human-derivates addition on hMSCs heterogeneity, observing that this aspect is not affected. These results suggested that the medium used during isolation is more crucial than the subsequent addition of derivates in the determination of cellular population fate.

The presented results display a better effect of human serum on hMSCs myogenic differentiation compared to PrP. Also, the readiness of serum isolation and availability contrasting with the industrious PrP isolation and with the different formulations (Shanbhag et al., 2017) make it a preferable choice.

In this study, we demonstrated the possibility to directly influence skeletal muscle-derived hMSCs isolation and behavior improving cell proliferation rate and myogenic efficacy by formulating specific culture media. Moreover, we have shown that by exploiting human blood derivates such as serum or PrP, one can achieve efficient and robust culture protocols for hMSCs expansion and differentiation that do not require animal derived supplements and can be easily translated into the clinical scenarios fulfilling GMP requirement.

## Data Availability Statement

The raw data supporting the conclusions of this article will be made available by the authors, without undue reservation.

## Ethics Statement

The studies involving human participants were reviewed and approved by IFO ethics committee. The patients/participants provided their written informed consent to participate in this study.

## Author Contributions

CG and MF conceived and designed the experiments. ST, CR, EF, SB, and CF carried out experiments. CG and SC supervised the project. ST and ER isolated and cultured human primary MSC besides critical reading. FR performed FACS analysis. JB provided muscle biopsies beside critical reading. MC, ST, EF, CR, and CG wrote the manuscript. All authors contributed to the article and approved the submitted version.

### Conflict of Interest

The authors declare that the research was conducted in the absence of any commercial or financial relationships that could be construed as a potential conflict of interest.
