# Supplementary material for: Skeletal Muscle-Derived Human Mesenchymal Stem Cells: Influence of Different Culture Conditions on Proliferative and Myogenic Capabilities
Source: Front Physiol. 2020 Sep 16;11:553198. doi: 10.3389/fphys.2020.553198 (PMC7526461; doi:10.3389/fphys.2020.553198)
Supplement: Supplementary file 1 [file Data_Sheet_1.pdf]

Supplementary Information

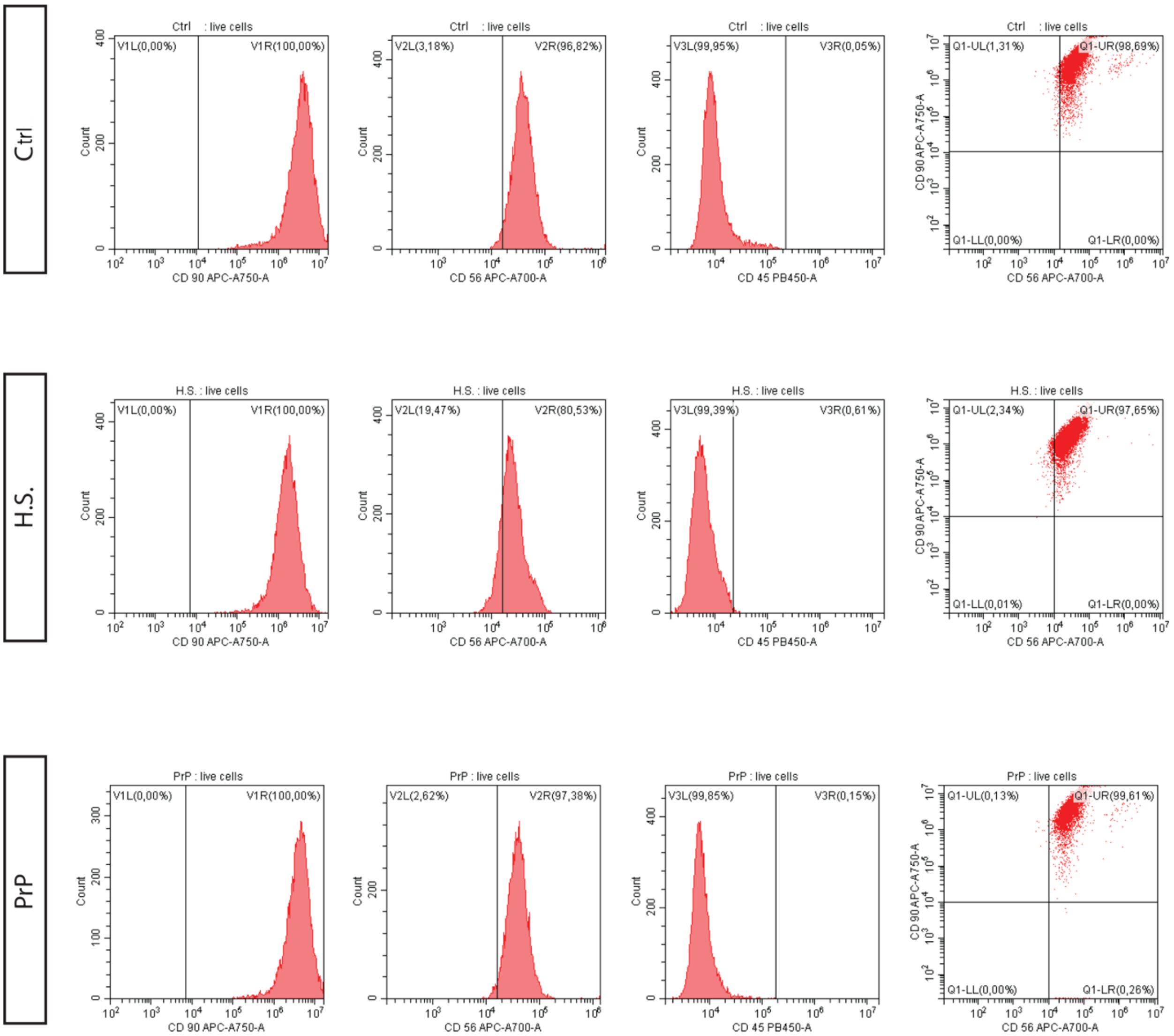

Supplementary Figure 1. hMSC characterization upon human serum and PrP exposure.

Flow cytometry analysis for CD56, CD90 and CD45 on hMSC cultured with standard supplement (FBS 20%) as control and high concentration (20%) of human serum (H.S.) and platelet rich plasma (1,5x10^6 platelets/ml) (PrP).
